# Glucokinase regulatory protein: a balancing act between glucose and lipid metabolism in NAFLD

**DOI:** 10.3389/fendo.2023.1247611

**Published:** 2023-08-29

**Authors:** Ziqi Zhang, Guang Ji, Meng Li

**Affiliations:** Institute of Digestive Diseases, Longhua Hospital, Shanghai University of Traditional Chinese Medicine, Shanghai, China

**Keywords:** glucokinase regulator, glucokinase regulatory protein, glucokinase, non-alcoholic fatty liver disease, and type 2 diabetes

## Abstract

Non-alcoholic fatty liver disease (NAFLD) is a common liver disease worldwide, affected by both genetics and environment. Type 2 diabetes (T2D) stands as an independent environmental risk factor that precipitates the onset of hepatic steatosis and accelerates its progression to severe stages of liver damage. Furthermore, the coexistence of T2D and NAFLD magnifies the risk of cardiovascular disease synergistically. However, the association between genetic susceptibility and metabolic risk factors in NAFLD remains incompletely understood. The glucokinase regulator gene (*GCKR*), responsible for encoding the glucokinase regulatory protein (GKRP), acts as a regulator and protector of the glucose-metabolizing enzyme glucokinase (GK) in the liver. Two common variants (rs1260326 and rs780094) within the *GCKR* gene have been associated with a lower risk for T2D but a higher risk for NAFLD. Recent studies underscore that T2D presence significantly amplifies the effect of the *GCKR* gene, thereby increasing the risk of NASH and fibrosis in NAFLD patients. In this review, we focus on the critical roles of GKRP in T2D and NAFLD, drawing upon insights from genetic and biological studies. Notably, prior attempts at drug development targeting GK with glucokinase activators (GKAs) have shown potential risks of augmented plasma triglycerides or NAFLD. Conversely, overexpression of GKRP in diabetic rats improved glucose tolerance without causing NAFLD, suggesting the crucial regulatory role of GKRP in maintaining hepatic glucose and lipid metabolism balance. Collectively, this review sheds new light on the complex interaction between genes and environment in NAFLD, focusing on the *GCKR* gene. By integrating evidence from genetics, biology, and drug development, we reassess the therapeutic potential of targeting GK or GKRP for metabolic disease treatment. Emerging evidence suggests that selectively activating GK or enhancing GK-GKRP binding may represent a holistic strategy for restoring glucose and lipid metabolic balance.

## Introduction

Non-alcoholic fatty liver disease (NAFLD) has become the most common chronic liver disease and is projected to become a leading cause of end-stage liver disease in the coming decades (1). NAFLD encompasses a range of histopathological types, from simple hepatic steatosis to the advanced form of non-alcoholic steatohepatitis (NASH) and fibrosis, which can eventually lead to cirrhosis and hepatocellular carcinoma (HCC) (2, 3). The prevalence of NAFLD has increased sharply in parallel with the global epidemics of obesity and type 2 diabetes (T2D). Currently, NAFLD affects approximately 32.4% of the population worldwide (4), with an even higher prevalence of up to 68% in individuals with T2D (5).

The relationship between NAFLD and T2D is bidirectional and complex. On the one hand, compelling evidence suggests that an accumulation of lipids in the liver is associated with insulin resistance and subsequent elevated risk of T2D development (6–9). On the other hand, T2D exacerbates the progression of NAFLD toward more advanced stages (10–13). In an updated meta-analysis, the global prevalence of NASH is 37.3% and the prevalence of advanced fibrosis is up to 17.0% in patients with T2D (5). Moreover, the simultaneous presence of T2D and NAFLD exponentially boosts the risk of cardiovascular disease, the primary mortality cause in NAFLD patients (14, 15). Considering that most NAFLD patients will eventually die from T2D and cardiovascular complications, targeting these interrelated conditions and adopting a holistic approach to treating metabolic diseases may hold significant promise.

Genetic insights provide a potent new approach for inferring and prioritizing drug candidates, a strategy that increases the success rate of drug development while identifying possible benefits (16). In recent years, with in-depth investigations of metabolism-related genes, genome-wide association studies (GWAS) have revealed the genetic basis for NAFLD. Various single nucleotide polymorphisms (SNPs) in lipid metabolism genes, such as patatin-like phospholipase domain-containing3 (*PNPLA3*), transmembrane 6 superfamily member 2 (*TM6SF2*), membrane-bound O-acyltransferase domain-containing 7 (*MBOAT7*), glucokinase regulator (*GCKR*) and hydroxysteroid 17-beta dehydrogenase 13 (*HSD17B13*), have been linked to the onset and progression of NAFLD (17–21). These key gene variants function in diverse pathways, encompassing lipid droplet remodeling, VLDL secretion, regulation of *de novo* lipogenesis (DNL), remodeling of phosphatidylinositol, and hepatic retinol availability (22). Emerging evidence suggests that in the presence of environmental risk factors, these genetic variants can further augment the risk of onset and progression of NAFLD (23, 24). Notably, the effect of *GCKR* polymorphism, in synergy with insulin resistance and T2D, in promoting the onset and progression of NAFLD has been increasingly recognized.

In 2011, the association between the *GCKR* polymorphism and NAFLD was identified for the first time through GWAS (25). Two common *GCKR* variants (rs1260326 and rs780094) have opposing effects in T2D and NAFLD: while they are associated with decreased levels of insulin resistance and a reduced risk of T2D, they increase the level of plasma triglycerides and the risk of NAFLD. Moreover, recent studies have revealed a strong correlation between *GCKR* variants and the development of NASH and fibrosis, particularly under conditions of metabolic stress (24, 26, 27). The *GCKR* gene encodes glucokinase regulator protein (GKRP), which functions as a switch and protector of glucokinase (GK) in the liver. Physiologically, in the fasting state, GKRP stores GK in the nucleus of hepatocytes in response to elevated postprandial glucose levels (28); In the postprandial state, GK dissociates from GKRP, leading to the release of GK into the cytoplasm and restoration of enzymatic activity, consequently stimulating glycolysis, glycogen synthesis, and DNL (29). Functional *GCKR* gene variants affect GKRP expression, localization, and sequestration ability, resulting in an easier dissociation of GK from GKRP and persistent stimulation of DNL. These findings imply that (I) overactivation of GK induced by *GCKR* variants causes an overload in the liver’s capacity to process glucose, increasing hepatic lipid accumulation; and (II) as a regulator and protector of GK, GKRP plays a role in maintaining glucose and lipid homeostasis, preventing liver damage from excessive metabolic substrates. Therefore, GKRP may serve as a potential therapeutic target for metabolic diseases such as T2D and NAFLD.

In this review, we focus on the specific role of the *GCKR* gene as well as the GKRP protein in the pathophysiology of NAFLD based on various genetic and biological studies. Therapeutic strategies aimed at GK or GKRP, such as glucokinase activators (GKAs) and GK-GKRP disruptors, are currently deemed beneficial for T2D management. However, considering the reduced long-term effectiveness of these drugs and their potential to elevate plasma triglycerides or induce fatty liver, an optimized approach may be warranted. Specifically, activating GK or enhancing GK-GKRP binding at selected moments, may represent a holistic strategy for restoring glucose and lipid metabolic balance.

## *GCKR* gene polymorphism and genetic susceptibility of NAFLD

The *GCKR* gene, which is located on chromosome 2, contains 19 exons and 18 introns and encodes GKRP. Two common variants in the *GCKR* gene, rs780094 (C>T) and rs1260326 (C>T), have been closely linked to a variety of metabolic diseases (25, 30–37). *GCKR* rs780094 is an SNP site in the noncoding region and is located in intron 16, while *GCKR* rs1260326 is located at site 446 in the exon 15 region and causes a replacement of proline with leucine (p.P446L). The two SNPs have been shown to have strong linkage disequilibrium (r^2^ = 0.93) (38) and the latter is a functional variant (39). Saxena et al. *(* 40) analyzed 386 731 common SNPs in 1464 T2D patients and 1467 matched controls to identify the association of the derived T allele of *GCKR* rs780094 with several metabolic phenotypes. These phenotypes included lower levels of FPG and insulin resistance, reduced risk of T2D, and higher levels of plasma triglycerides. Orho-Melander et al. (38) verified that *GCKR* rs780094 was associated with higher plasma triglycerides and lower FPG levels, and the missense variant rs1260326 (C>T p.P446L) showed the strongest association with plasma triglyceride levels in 12 independent cohorts.

Liver fat accumulation is a hallmark feature of NAFLD. To confirm the correlation between *GCKR* gene polymorphisms and NAFLD, Santoro et al. (21) evaluated the impact of *GCKR* rs1260326 on hepatic fat content, triglycerides, and lipoprotein levels in a population of 455 obese children and adolescents. Their findings indicated that *GCKR* rs1260326 was associated with liver fat accumulation and elevated plasma VLDL, with homozygote carriers of the *GCKR* minor allele accruing 180% more hepatic fat compared to homozygote carriers of the *GCKR* major allele. Additionally, the researchers revealed a synergistic effect between genetic variants in *GCKR* and *PNPLA3* that increased susceptibility to NAFLD in obese adolescents. A GWAS conducted by Speliotes et al. (25) on 7,176 individuals from multiple centers established that *GCKR* rs780094 was associated with NAFLD. The *GCKR* risk allele that increased liver fat content was founded to be associated with lower FPG, fasting insulin, and homeostatic model assessment for insulin resistance (HOMA-IR), but higher plasma low-density lipoprotein cholesterol (LDL-C), triglycerides, and 2-h postprandial glucose levels. These findings have received confirmation from various population studies conducted across different regions (41–45). *GCKR* gene polymorphisms may also influence the histological progression of NAFLD (25, 46–48). To clarify whether hepatic steatosis-related *GCKR* gene variants correlate with the histological progression of NAFLD, Speliotes et al. (25) genotyped 592 patients with biopsy-confirmed NAFLD from the NASH Clinical Research Network. Their findings indicated that the *GCKR* rs780094 (effect allele: T) was associated with more severe NASH/fibrosis in NAFLD patients. In addition, a multivariate logistic regression analysis was performed in a cohort of 366 patients with NAFLD. This analysis revealed that the *GCKR* rs780094 variant (C>T) was independently correlated with NAFLD activity score (NAS ≥ 5), even after adjusting for the influence of the *PNPLA3* gene (46). Anstee et al. (49) conducted the largest GWAS to date that included the histological features of NAFLD, encompassing the entire disease spectrum from steatosis to cirrhosis. The results implied rs1260326 T-variant carriage increased NAFLD, NASH and advanced fibrosis risk.

Nevertheless, some studies have reported inconsistent results (**Table 1**). For instance, Ajmera et al. (51) assessed the effect of several genetic variants on advanced fibrosis in NAFLD, defined as liver stiffness ≥ 3.63 kPa, using magnetic resonance elastography. Although the risk allele variants of *GCKR* were associated with increased liver stiffness, the association did not reach statistical significance. Similarly, Holmer et al. (50) collected DNA samples from 546 patients with NAFLD diagnosed with advanced fibrosis by liver biopsy or elastography. When compared to matched healthy controls, *GCKR* gene variants were not associated with NASH or severe liver disease (hepatic decompensation or HCC) after adjusting for factors including age, gender, body mass index (BMI), and the risk ratio for T2D using Cox regression. Given that disease activity in susceptible individuals may fluctuate depending on environmental triggers (52), it may be crucial to further evaluate the impact of *GCKR* effect alleles on the histological progression of NAFLD in populations with impaired metabolism, particularly diabetes.

**Table 1:** List of common *GCKR* variants associated with hepatic steatosis, NASH and fibrosis.

Gene	Study	Variant	Diagnosis	Case number	Case control	Hepatic steatosis	NASH	Fibrosis
*GCKR*	Holmer (2022) (50)	rs1260326 C>T	TE / Biopsy	546	5396		–	–
	Ajmera (2021) (51)	risk allele: T	MRE	264				–
	Anstee (2020) (49)	rs1260326 C>T	Biopsy	1483	17781	+	+	+
	Hudert (2019) (48)	rs780094 C>T	Biopsy	70	200	+		+
	Tan (2014) (47)	rs1260326 C>T	Biopsy	144	198	+	+	+
	Petta (2014) (46)	rs780094 C>T	Biopsy	366		+	+	+
	Santoro (2012) (21)	rs1260326 C>T	MRI	142		+		
	Speliotes (2011) (25)	rs780094 C>T	CT / Biopsy	7176		+	+	+

Te, Transient elastography; MRE, Magnetic resonance elastography; MRI, Magnetic resonance imaging; CT, Computed tomography; NASH, non-alcoholic steatohepatitis; Plus sign, There is evidence of an association between this variant and disease susceptibility; Minus sign, No association was found between this variant and disease susceptibility.

## *GCKR* as the nexus of genetics and metabolism in NAFLD

The development and progression of NAFLD involve a complex interplay between genetic and environmental factors. However, there are few convincing examples of NAFLD risk genes interacting with T2D. This paucity may be partially attributed to the fact that most genes implicated in NAFLD are more closely associated with lipid rather than glucose metabolism. The phenotypic manifestations of individual gene mutations could be amplified by interactions between genes and the environment, with the *GCKR* gene exhibiting strong synergistic effects alongside metabolic factors, especially insulin resistance and diabetes.

To identify metabolic risk factors that interact with genetic variants for NAFLD, Barata et al. (26) probed associations between common genetic variants and key metabolic indicators including blood glucose, insulin, insulin resistance, triglycerides, LDL-C, high-density lipoprotein cholesterol (HDL-C), BMI, and waist-to-hip ratio. The study showed that the *GCKR* rs780094 significantly interacted with insulin resistance, increasing the susceptibility of nondiabetic individuals to NAFLD. Further evidence from Stender et al. (24) demonstrated that obesity markedly amplified the genetic risk of NAFLD associated with *GCKR* rs1260326. Thus, obesity and genotype have a synergistic and promotive action on the entire spectrum of NAFLD from simple steatosis to hepatic inflammation to cirrhosis.

Critically, as an essential gene in the glucose metabolism pathway, the contribution of the *GCKR* gene to NASH/fibrosis is highly dependent on diabetes status (27, 53). In T2D patients, the association between *GCKR* rs1260326 (minor allele: T) and plasma triglycerides and HDL-C was stronger than in healthy controls, suggesting that glucose metabolism may influence the strength of the association between rs1260326 and plasma lipids (54). Kimura et al. (27) devised a pooled human organoid panel of NASH to investigate the effect of metabolic status on genotype-phenotype associations. Their population-based phenotypic analysis predicted that *GCKR* rs1260326 (C>T) serves as a key genetic factor for NASH under insulin resistance conditions. *GCKR* rs1260326 was associated with liver fat accumulation phenotype independently of *PNPLA3* rs738409 in the absence of exogenous lipid induction. The study further assessed the interaction between genes and metabolic status. When glycated hemoglobin (HbA1c) values were within the normal range (< 5.7%), alanine transaminase, NAS, lobular inflammation, and SAF (steatosis, activity, and fibrosis) scores were significantly better in patients carrying the *GCKR* TT variant than those carrying the CC variant. Conversely, when HbA1c was > 6.4%, the *GCKR* TT group had higher scores than the *GCKR* CC group, indicating a deteriorating pathology. *GCKR* rs1260326 (minor allele: T) may prevent fibrosis in the nondiabetic state, but increases disease severity in the diabetic state, promoting NAFLD histological progression by affecting triglyceride levels, insulin resistance, DNL and mitochondrial function. In summary, the phenotypic effects of *GCKR* gene mutations can be amplified by the interaction of genetic and metabolism factors, positioning the *GCKR* gene as a key mediator linking metabolic damage to inflammation and fibrosis in fatty liver disease.

**Figure 1:**
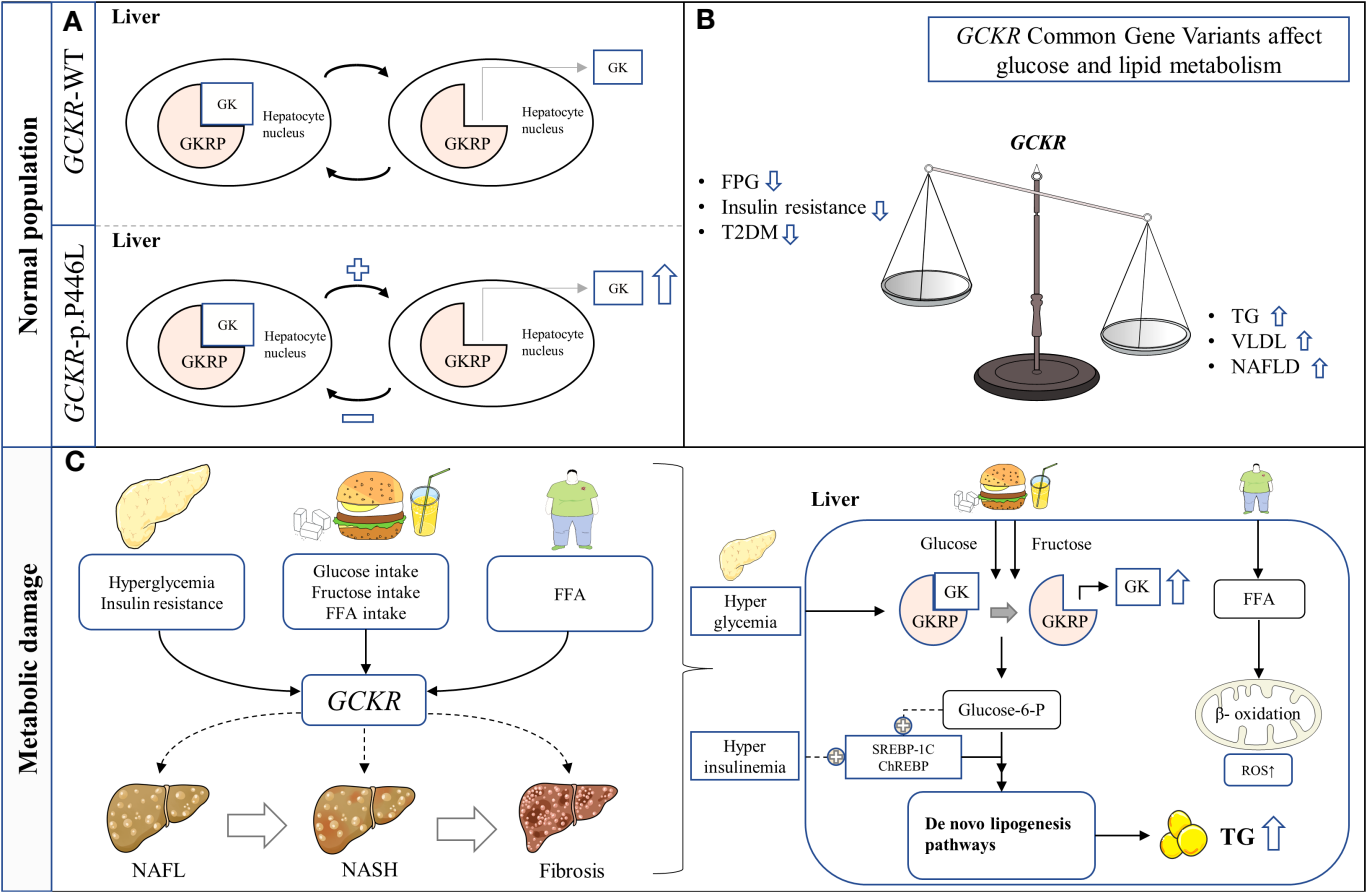
Schematic representation of hypothesized mechanism linking the *GCKR* rs1260326 p.P446L genetic variant with NAFLD in hepatocytes. **(A)** The presence of *GCKR* p.P446L leads to the easier dissociation of GK from GKRP and activation. **(B)** *GCKR* reduces FPG, insulin resistance, and the risk of T2D, but increases serum triglyceride levels and susceptibility to NAFLD. **(C)** *GCKR* acts synergistically with environmental risk factors to promote the histological progression of NAFLD. FFA, Free fatty acid; FPG, Fasting plasma glucose; GK, Glucokinase; GKRP, Glucokinase regulatory protein; NAFL, Nonalcoholic fatty liver; NASH, Nonalcoholic steatohepatitis; ROS, Reactive oxygen species; T2DM, Type 2 diabetes mellitus; TG, Triglyceride.

The association between the *GCKR* gene variants and metabolic status is associated with the consumption of specific diets (55–58). Given the pivotal role of GKRP in glucose and lipid metabolism, it is important to determine whether the effects of *GCKR* gene variants on liver fat content depend on high carbohydrate intake. Since high GK activity is expected to increase hepatic glucose uptake, it is possible that high carbohydrates (such as glucose and fructose) and high fat intake will further increase hepatic lipogenesis and exacerbate NAFLD (59–61). Evidence on this topic remains limited. In conclusion, these findings highlight the importance of considering gene-environment interactions when studying the pathological mechanisms of NAFLD (**Figure 1**).

## Characteristics and biological functions of GKRP

GKRP is a 68-kDa protein encoded by the *GCKR* gene and is mainly expressed in the liver. As an endogenous competitive inhibitor of GK, it plays a significant role in regulating GK activity (28, 62–65). GK, which is an essential component of the glucose sensing system (66), controls the rate of glucose uptake and glycogen synthesis in the liver: it catalyzes the conversion of glucose to glucose-6-phosphate (G6P), which is the first reaction in hepatic glucose metabolism (67). In the fasting state, GK remains inactive and binds to GKRP in the nucleus of hepatocytes as a reserve response to postprandial blood glucose elevation (28); In the postprandial state, GK dissociates from GKRP, leading to the release of GK into the cytoplasm and restoration of enzymatic activity, consequently stimulating glycolysis, glycogen synthesis, and DNL (29, 68–70).

GKRP possesses a protective effect on GK. Since GKRP is an inhibitor of GK, it was initially thought that the reduction or complete absence of GKRP in the liver would lead to higher GK activity. However, contrary to expectations, with the knockout or knockdown of GKRP, GK expression and activity decrease simultaneously in the liver (71, 72). In both mouse and human GKRP, the P446>L substitution has also been observed to reduce GKRP protein expression and lead to decreased hepatic GK levels (73). This suggests that GKRP has a stabilizing effect on GK, and that any knockdown or mutation of GKRP could dimmish this effect, leading to lower GK expression in hepatocytes. Without sufficient GK in the nucleus, hepatocytes cannot mobilize enough GK into the cytoplasm in response to changes in glucose levels. Due to the synchronous decrease in both total GK protein levels and nucleus reserves, hepatocytes from P446L mice had lower metabolic rates at elevated glucose concentrations, underscoring the acute regulatory role of GKRP in response to elevated glucose (73). In homozygote knockout mice (GKRP^-/-^), the disruption of regulatory function and the corresponding reduction in GK activity leads to impaired glucose homeostasis, as demonstrated by reduced glycogen levels, elevated expression of *PEPCK* gene encoding the gluconeogenic enzyme, and impaired glucose disposal under a glucose challenge (71). Notably, unlike the elevated blood glucose observed in GKRP^-/-^ on a high-sucrose/high-fat diet (71), the P446L mice showed lower blood glucose levels when fed a sugar containing diet (73). The discrepancy might be ascribed to the modest retention of GKRP still present in the P446L mice. Overall, severe GKRP deficiency is associated with the development of diabetes-related phenotypes, particularly in the presence of the risk factors such as high-fat, or high-carbohydrate diets (71).

GK affects the subcellular localization of GKRP. GKRP is predominantly, but not exclusively present in the nucleus of hepatocytes. It can shuttle between the nucleus and cytoplasm (74–77). In rats with reduced or lacking levels of GK protein, GKRP was abnormally localized to the cytoplasm of hepatocytes (78). Alternatively, GKRP also determines the subcellular localization of GK in hepatocytes (76, 77). The sequestration of GK in the nucleus is highly dependent on the presence of GKRP, and in GKRP^−/−^ mice, GK is only present in the cytoplasm of hepatocytes (71). However, a recent study has indicated that in hepatocytes and other cell models, GK overexpression leads to an increase in the nucleus sequestration of GKRP. This finding suggests that nuclear GKRP sequestration also depends on the levels of GK protein (73). By sequestrating GK in the nucleus, GKRP helps minimize hepatic glucose phosphorylation during the fasting state and allows sufficient GK to be mobilized into the cytoplasm for glucose metabolism in the postprandial state. This regulatory mechanism enables the liver to effectively responds to fluctuations in blood glucose concentrations during the feeding-fasting cycles, helping to maintain blood glucose concentrations within the normal physiological range.

Multiple metabolic and hormonal conditions regulate the nucleus-cytoplasmic translocation of hepatic GK (29, 76). GK plays an essential role in the hepatic glucose sensing system, which induces an adaptive response to balance hepatic glucose consumption and storage in the liver (66). High glucose concentrations disrupt GK-GKRP binding, translocate GK to the cytoplasm, and induce a conformational shift of GK to a high affinity for glucose. Small molecule glucokinase activators (GKAs) can promote GK activation by binding to the variable conformation site of GK and stabilizing the high affinity conformation (79, 80). Fructose plays a key role in regulating the binding of the GK-GKRP complex, with fructose-1-phosphate (F1P) inhibiting the binding while fructose-6-phosphate (F6P) enhances it (64, 81). F1P, a phosphorylated form of fructose, disrupts the GK-GKRP complex and promotes GK translocation to the cytoplasm. Fructose promotes glucose phosphorylation more strongly and rapidly than glucose, and glucose and fructose promote GK translocation to the cytoplasm in a synergistic manner (74, 82). Consequently, feeding catalytic amounts of fructose has been shown to increase hepatic glucose uptake and glycogen storage, improve glucose tolerance (83, 84), and restore the ability of blood glucose to inhibit hepatic gluconeogenesis (85). These benefits may only be short-term. F6P stabilizes the GK-GKRP complex. GKRP can be regarded as a sequestration protein that inhibits GK and ensures its removal from gluconeogenesis. This mechanism prevents ineffective glucose cycling. Hormones also play a significant role in the regulation of GK translocation. Glucagon is predicted to promote GK sequestration in the nucleus, under glucose and fructose induction, glucagon partially reverses the adaptive translocation of GK to the cytoplasm (86). In contrast, elevated circulating insulin has an enhancing effect on GK translocation and upregulates GK mRNA expression, promoting glucose uptake in the liver (87).

It is worth noting the relative expression between GK and GKRP. As GKRP expression increases, the affinity of the GK-GKRP complex for F6P increases, while that for F1P decreases (62, 88). Overexpression of GKRP with recombinant adenovirus has been shown to inhibit glucose phosphorylation, glycolysis, and glycogen synthesis at various concentrations of glucose and sorbitol, and reduce the affinity of GK translocation for glucose. At high glucose concentrations (35 mmol/L), but not at low concentrations (7.5 mmol/L), a moderate increase in GK expression was associated with a decrease in the GKRP control coefficient on glycogen synthesis, indicating that the GK-GKRP ratio affects the affinity of hepatocytes for glucose (88). These findings suggest that adaptive changes in GK/GKRP ratio safeguard efficient glucose uptake and protect the liver from damage caused by an excess of the metabolic substrate.

In conclusion, GKRP is a crucial component of the GK translocation machinery in the liver, regulating GK activity in response to metabolic alterations. This mechanism confers glucose-dependent responsiveness and sensitivity in hepatocytes, allowing for effective glucose uptake across a wide range of glucose concentrations (76, 88). Understanding the factors underlying the regulation of GK and GKRP is critical for developing strategies to prevent and treat metabolic disorders including diabetes and NAFLD.

## Hypothetical mechanism of GKRP in NAFLD

Genetic and biological studies have placed GKRP at the crossroads of hepatic triglycerides and glucose metabolism. Beer et al. (39) revealed the genetic mechanism underlying this association: *GCKR* rs1260326 (C>T p.P446L) diminishes the ability of GK-GKRP to respond to F6P. Since F6P is a natural facilitator of GK-GKRP binding, GK is more likely to dissociate from GKRP when GKRP-P446L is present. This is predicted to enhance glycolytic flux, thereby increasing glucose uptake by the liver (39). In addition, *GCKR* variant p.P446L influences cellular localization, ability to interact with GK, and kinetic activity of the encoded proteins (89).

Indeed, *GCKR* is one of the most pleiotropic genes, which is associated with metabolites of carbohydrate, fatty acid, purine, amino acid, and lipid metabolism. Such metabolites may be implicated in major biological pathways associated with NAFLD progressions, such as inflammation, oxidative stress, and lipid metabolism (**Table 2**). Impaired carbohydrate metabolism is a major contributor to NAFLD pathogenesis. The assumed hypothesis for the raised blood and liver triglycerides and lower blood glucose associated with the *GCKR* locus is that the *GCKR* P446>L impairs GK binding to GKRP, and thereby promotes hepatic conversion of glucose to triglyceride through the uninhibited GK. This may explain the reduction in fasting plasma glucose and insulin levels (39).

**Table 2:** *GCKR* common gene variants in relation to metabolomics.

Group	Metabolite	Effect	Refs
Carbohydrate metabolism	Fasting plasma glucose	↓	(38, 40, 90–92)
	Postprandial blood glucose	↑	(25, 93)
	Insulin	↓	(25, 40, 91–93)
	Pyruvate	↑	(94)
	Lactate	↑	(95)
Fatty acid metabolism	Fatty acids	↑	(94, 96)
	Glycerol	↑	(96)
	β-OH butyrate	↓	(97)
Purine metabolism	Urate	↑	(98–100)
Amino acid metabolism	Alanine	↑	(94, 101)
	Glutamine	↓	(101)
	Tyrosine	↓	(101)
	Histidine	↓	(101)
	Isoleucine	↑	(94, 101)
	Leucine	↑	(94, 101)
Lipid metabolism	Triglycerides	↑	(21, 38, 40, 91, 102)
	Total cholesterol	↑	(98, 100, 103)
	VLDL	↑	(21, 94, 96, 102)
	VLDL particles	↑	(94)
	HDL	↓	(92, 93, 104)
	LDL	↑	(25, 94, 98, 100, 102)
	Apolipoprotein B	↑	(38, 94, 96)
	Apolipoprotein CII	↑	(96)
	Apolipoprotein CIII	↑	(96)
Inflammation and immunity	CRP	↑	(38, 93, 98, 102, 105)
	Alpha-1 antitrypsin	↓	(106)
	Complement C3	↑	(107)
Liver enzyme	ALT	↑	(108)
	GGT	↑	(94)
Others	FGF21	↑	(109)
	sE-selectin	↑	(110)
	Follistatin	↑	(111)
	Butyrylcholinesterase	↑	(112)
	Serum calcium	↑	(113)
	Factor VII	↑	(114, 115)
	Factor XI	↑	(116)
	Protein C	↑	(117)
	Creatinine	↓	(98, 100)
	Serum albumin	↑	(100, 113)
	Urine albumin	↑	(98, 100)
	Cystatin-C	↓	(98)

VLDL, Very-low-density lipoprotein; HDL, High-density lipoprotein; LDL, Low-density lipoprotein; CRP, C-reactive protein; ALT, Alanine transferase; GGT, Gamma-glutamyltransferase; FGF21, Fibroblast growth factor 21; Up arrow, indicates an increase; Down arrow, indicates a decrease.

However, the P446>L substitution compromises the protein expressivity of GKRP and nucleus sequestration of GK (73), resulting in an inability of hepatocytes to mobilize sufficient GK in response to changes in glucose levels, manifesting as impaired postprandial glucose tolerance (25, 93). This is supported by recent evidence that hepatocytes from P446L mice have a lower metabolic rate at elevated glucose but not at physiological glucose concentration (73). GK catalyzes the conversion of glucose into G6P, and an increase in G6P can cause phosphate depletion and increased uric acid production (118). It is believed that impaired hepatic phosphate homeostasis contributes to hepatic glucose production and lipogenesis (119). Carbohydrate metabolism dysfunction leads to consequent aberrant homeostasis of intermediary metabolism, and increased glucose metabolism via the glycolytic pathway ultimately leads to elevated malonyl-CoA. Malonyl-CoA can promote NAFLD via different pathways (I): serving as the substrate for DNL, resulting in steatosis and insulin resistance; and (II) limiting fatty acid β-oxidation by inhibiting the mitochondrial fatty acid transporter carnitine palmitoyl transferase-1 (120), which further promotes fatty acid accumulation.

DNL and inhibition of fatty acid β-oxidation make important contributions to the development of NAFLD (121–123). Stable isotope studies have reported an association between *GCKR* risk alleles and DNL, and homozygote carriers of *GCKR* rs1260326 (minor allele: T) have exhibited higher levels of fasting DNL but lower levels of DNL after carbohydrate loading (124). Thus, the contribution of *GCKR* risk alleles to hepatic fat accumulation may largely depend on the enhanced synthesis of the DNL pathway in the fasting state. Alternatively, recent studies have reported an association of *GCKR* with mitochondrial dysfunction (27). Transcriptomic, metabolomic, and pharmacological analyses indicate significant mitochondrial dysfunction incurred by *GCKR* rs1260326 (27). The human organoid study has provided evidence that the *GCKR* rs1260326 variant results in a reduced mitochondrial oxygen consumption rate and consistently enhanced reactive oxygen species levels (27). When the liver is overloaded with the ability to process major metabolic energy substrates, accumulation of toxic lipid species occurs, promoting the development and progression of NAFLD (123).

**Figure 2:**
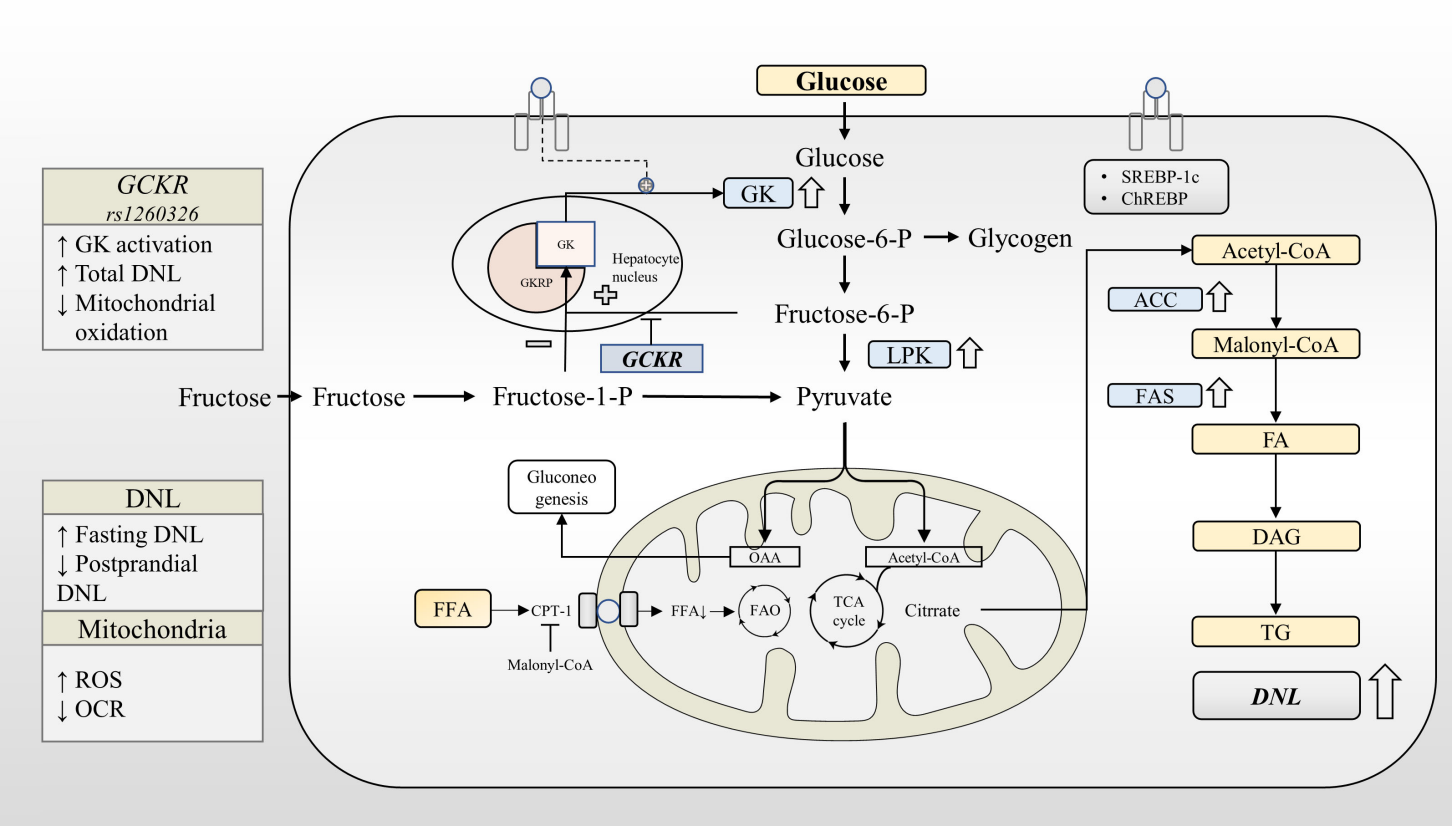
*GCKR* rs1260326 regulates hepatic glucose and lipid metabolism through increasing glucokinase activation. Under normal physiological conditions, glucose and insulin synergistically promote GK translocation and activate the expression of lipogenesis-related genes through GK-dependent transcription factors. GKRP binds to GK more strongly in the presence of F6P and less strongly in the presence of F1P. The *GCKR* gene variant reduces the sensitivity of the GK-GKRP complex to F6P, making it easier for GK to dissociate from GKRP. Under the influence of *GCKR* gene variant, GK is activated at a lower glycemic threshold. This leads to increased glycogen synthesis, glycolytic flux, and activation of the DNL pathway. *GCKR* gene variant reduces fasting glucose levels at the expense of increased liver fat content. ACC, Acetyl-CoA carboxylase; CPT-1, Carnitine palmitoyltransferase-1; DAG, Diacylglycerol; DAGT, Diacylglycerol acyl transferase; DNL, *de novo* lipogenesis; FA, Fatty acid; FAO, Fatty acid oxidation; FAS, Fatty acid synthase; GK, Glucokinase; GKRP, Glucokinase regulatory protein; LPK, Liver-type pyruvate kinase; OCR, Oxygen consumption rates; ROS, Reactive oxygen species; TG, triglyceride.

Finally, recent genetic studies have found that common genetic variants in *GCKR* are associated with varying levels of CRP, follistatin, fibroblast growth factor 21, sE-selectin, etc, and the association between these factors and NAFLD has been reported (110, 111, 125–127). It should be noted that variants in other genes near the *GCKR* locus, which are in linkage disequilibrium, may have additional synergistic effects on metabolites. In summary, *GCKR* has a metabolic signature that closely resembles overall NAFLD, suggesting that GKRP may be a therapeutic target capable of improving intermediary metabolism. The association of *GCKR* with metabolites further revealed a possible biological role of GKRP in the pathogenesis of NAFLD (**Figure 2**).

## Lessons from GKAs and GK-GKRP disruptors

Over the past two decades, GK has emerged as a promising target for diabetes treatment (128). Dozens of GKAs, including pancreatic-hepatic dual activators and hepatoselective activators, have been evaluated and developed. However, most GKAs failed in their early development stages due to issues with hypoglycemia and a lack of long-term efficacy (129–132). Additionally, some GKAs were associated with elevated triglycerides and an increased risk of NAFLD (130–133). The discovery of adverse reactions to early drug candidates has spurred the development of next-generation activators designed to mitigate these risks. From the success or failure of GK activator development, there are several valuable experiences.

To mitigate the hypoglycemia risk of GKAs, several research groups have focused on designing "partial activators". These increase the enzymatic activity of GK without excessively reducing the Michaelis constant (K_m_) for glucose. Among these, AZD1656 is a leading candidate. However, during a phase 2 clinical trial, AZD1656, administered twice daily with meals for six months, was discontinued due to a progressive loss of its hypoglycemic effect and elevated plasma triglycerides (131). Considering the short half-life time of AZD1656, twice daily administration is often recommended to prolong GK activation (134, 135), but this near-round-the-clock GK activation can increase the risk of NAFLD.

Other research groups have focused on hepatoselective activators, which aim to reduce the risk of hypoglycemia by avoiding insulin secretion. However, the potential risk of hyperlipidemia and fatty liver still restricts their development, which may stem from inherent limitations within the targets themselves (133, 136, 137). Overexpression of GK in the liver is associated with hepatic fat accumulation. Using recombinant adenovirus to overexpress GK in the liver of normal rats can lower both blood glucose and circulating insulin levels, but cause a sharp increase in circulating triglycerides and free fatty acid levels (138). In an analysis of patients with liver biopsies, a positive association was observed between hepatic GK mRNA expression and triglyceride levels, the expression of lipogenic genes, as well as the DNL index (139). A recent study delving into the long-term effects of hepatic GK overexpression on glucose homeostasis revealed that GK overexpression did not prevent insulin resistance induced by a high-fat diet. Instead, chronic GK overexpression amplified hepatic lipogenesis and circulating lipids, contributing to insulin resistance and compromised glucose tolerance (59). In a study assessing potential lipogenic risks linked to oral GKAs, all three types of GKAs (despite different structures) induced hepatic steatosis in *db/db* mice. This supports the notion that the effects of GKAs on hepatic steatosis are mediated through drug-target effects (133). These findings suggest that differences in GK expression, activity, and the timing of activation can lead to completely different effects on glucose and lipid metabolism. In addition, given the indirect activating effect on GK, small-molecule GK-GKRP disruptors have also raised concerns about the risk of elevated plasma triglycerides and fatty liver (140). The essence of GK-GKRP disruptors is to reverse the inhibitory effect of GKRP on GK activity and reduce the nuclear sequestration of GK, resulting in an increase in the amount of cytosolic GK without altering the inherent kinetics of the enzyme (140–142). Preclinical studies have shown that blood glucose-lowering effect of GK-GKRP disruptors is restricted to diabetic animals and not observed in normoglycemic ones, indicating an effective strategy to reduce the risk of hypoglycemia. Plasma triglycerides were also found to be unchanged in the ZDF rats after treatment with these disruptors (141). It's important to acknowledge that all the current evidence on GK-GKRP disruptors is based on short-term preclinical studies (142). Although present evidence does not suggest an increased risk of triglyceride levels with GK-GKRP disruptors, a comprehensive evaluation of the long-term effects of GK-GKRP disruptors on lipid metabolism remains crucial.

Preserving the physiological regulation of GK by GKRP is considered crucial to avoid lipid metabolism disorders. GKAs that do not disrupt GK-GKRP binding, like the hepatoselective activator TTP399, seem to avoid liver lipid accumulation (143). Four weeks of TTP399 treatment in diabetic mice resulted in reduced plasma and liver TG concentrations. In a phase 2 clinical trial, six months of TTP399 treatment substantially improved glycemic control in T2D patients, without causing hypoglycemia or hyperlipidemia. Further studies conducted in rat hepatocytes supported that the physiological regulation of GK by GKRP is maintained in the presence of TTP399, ensuring that TTP399 increases GK activity only during hyperglycemia (143). Therefore, GKRP-dependent metabolic flexibility is necessary for balancing hepatic glucose and lipid metabolism.

## Novel strategies for targeting GK or GKRP for the treatment of T2D and NAFLD

The novel GKAs, TTP399 and dorzagliatin (143, 144), seem to have overcome the side effects of lipid metabolic disorder. However, the specific role of GKAs in metabolic diseases remains shrouded by several unresolved questions: (i) The long-term efficacy of GKAs is under scrutiny. Previous GKAs have experienced a decline in long-term efficacy for reasons yet to be fully understood (128). Data beyond three months for dorzagliatin and six months for TTP399 is still lacking. (ii) Considerations of long-term safety and additional therapeutic benefits are essential. An ideal novel drug should address chronic hyperglycemia and its long-term complications, particularly cardiovascular disease. However, the effects of GKAs on cardiovascular disease seem to be at best neutral. (iii) Determining the optimal mode of drug administration remains essential. Interestingly, a recent study found that AZD1656, previously associated with an increased risk of fatty liver, improved glycemia, hepatic steatosis, and inflammation by chronotherapy (145).

In the study involving obese Zucker rats, different exposure patterns of AZD1656 were evaluated, encompassing continuous 24-hour exposure or timed exposure during feeding or fasting periods. Continuous 24-hour therapeutic exposure to AZD1656 improved glycemic control in obese Zucker rats but also led to hepatic steatosis and inflammation. Conversely, intermittent AZD1656 treatment, timed to coincide with the feeding period, reversed hepatic steatosis and inflammation, reduced fibrosis marker expression, and improved glycemic. As previously mentioned, the contribution of common *GCKR* gene variants to NAFLD may primarily originate from hyperactivation of hepatic GK during fasting (39, 124). Chronotherapy conferred additional advantages to AZD1656, facilitating improvements in hepatic steatosis, metabolic flexibility, and insulin sensitivity (145). The question of whether chronotherapy could apply to other GKAs remains to be explored.

Given the adverse consequences of over-activation of hepatic GK, some researchers have proposed that inhibiting hepatic GK may have potential metabolic benefits (146, 147). However, more important is how to strike a balance between the activation and inhibition. As a natural regulator for GK, GKRP emerges as a prominent candidate for this strategy. Overexpression of GKRP in mice induced to develop diabetes through a high-fat diet did not exacerbate the condition (148). Instead, all pre-existing symptoms disappeared and FPG levels decreased to a level similar to that of nondiabetic mice, suggesting that increased GKRP expression can correct impaired glucose metabolism. In the absence of hepatic steatosis or increased plasma triglyceride, these mice exhibited lower leptin levels, reduced body weight, and higher insulin sensitivity. Overexpression of GKRP decreased hepatic GK activity but increased nucleus GK stores, suggesting a more efficient and flexible manner of GK in metabolizing blood glucose. *In vivo*, compared to GK overexpression alone, simultaneous overexpression of both GK and GKRP significantly increased hepatic GK protein levels and activity in HepG2 cells (148). Therefore, it is reasonable to speculate that GKRP has the potential to improve metabolic dysfunction-associated fatty liver disease by restoring GK-dependent metabolic flexibility and effectiveness.

Despite the uncertainties surrounding this strategy, multiple lines of evidence provide support. Genetic studies suggest that missense variants of *GCKR* increase the risk of NAFLD and cardiovascular disease, hinting at potential benefits of enhanced GK-GKRP binding. Considering the contradictory effects of GKRP on glucose and lipid metabolism, enhancing GK-GKRP binding may raise concerns about the risk of hyperglycemia. Nevertheless, GKRP overexpression in T2D mice has shown long-term beneficial effects on glucose metabolism (148). Moreover, GKRP overexpression improved insulin sensitivity in T2D mice, which is central to the onset and progression of NAFLD. Additionally, insights gained from chronotherapy provide a viable paradigm for strategies aiming to balance glucose and lipid metabolism through GK or GKRP. By balance of glucose and lipid metabolism, it’s possible to see simultaneous improvement in T2D, NAFLD, and perhaps even cardiovascular disease.

## Conclusions

NAFLD is a multifactorial disease caused by the interaction between genetic susceptibility and environmental risk factors. Emerging evidence suggests that *GCKR* gene polymorphisms contribute to the pathogenesis and progression of NAFLD through synergistic effects with metabolic risk factors. Based on evidence from genetics, biology, and drug development, excessive activation of hepatic GK may be detrimental to glucose and lipid metabolism: (I) constant activation of hepatic GK may lead to progressive deterioration of GK function, resulting in abnormal glucose metabolism; and (II) excessive glucose uptake stimulates lipogenesis through multiple pathways, leading to the development and progression of NAFLD. Conversely, evidence from chronotherapy and GKRP overexpression suggest that balancing GK activation and restriction may be a more comprehensive therapeutic strategy. GKRP plays a dual role in GK protection and regulation. Overexpression of GKRP may help to protect GK function, enhance postprandial glucose metabolism, ultimately help to reverse chronic hyperglycemia, and restore metabolic flexibility. By balancing glucose and lipid metabolism, this strategy may further reduce the occurrence and progression of NAFLD.

## Author contributions

ZQZ performed the literature review and wrote the manuscript. ML and GJ conceptualized the idea, and critically reviewed and revised the manuscript. All authors contributed to the article and approved the submitted version.
